# Extremely Low Vapor‐Pressure Data as Access to PC‐SAFT Parameter Estimation for Ionic Liquids and Modeling of Precursor Solubility in Ionic Liquids

**DOI:** 10.1002/open.202000258

**Published:** 2021-01-25

**Authors:** Mark Bülow, Moritz Greive, Dzmitry H. Zaitsau, Sergey P. Verevkin, Christoph Held

**Affiliations:** ^1^ Laboratory of Thermodynamics Department of Biochemical and Chemical Engineering TU Dortmund Emil-Figge Str. 70 44277 Dortmund Germany; ^2^ Department of Physical Chemistry University of Rostock 18059 Rostock Germany; ^3^ Department of Physical Chemistry Kazan Federal University 420008 Kazan Russia

**Keywords:** activity coefficients, density, ionic liquids, phase equilibrium, thermodynamics

## Abstract

Precursor solubility is a crucial factor in industrial applications, dominating the outcome of reactions and purification steps. The outcome and success of thermodynamic modelling of this industrially important property with equations of states, such as Perturbed‐Chain Statistical Associating Fluid Theory (PC‐SAFT), vastly depends on the quality of the pure‐component parameters. The pure‐component parameters for low‐volatile compounds such as ionic liquids (ILs) have been commonly estimated using mixture properties, e. g. the osmotic pressure of aqueous solutions. This leads to parameters that depend on the solvent, and transferability to other mixtures often causes poor modeling results. Mixture‐independent experimental properties would be a more suitable basis for the parameter estimation offering a way to universal parameter sets. Model parameters for ILs are available in the literature [10.1016/j.fluid.2012.05.029], but they were estimated using pure‐IL density data. The present work focuses on a step towards a more universal estimation strategy that includes new experimental vapor‐pressure data of the pure IL. ILs exhibit an almost negligible vapor pressure in magnitude of usually 10^−5^ Pa even at elevated temperatures. In this work, such vapor‐pressure data of a series of 1‐ethyl‐3‐methyl‐imidazolium‐based [C_2_mim]‐ILs with various IL‐anions (e. g. tetrafluoroborate [BF_4_]^−^, hexafluorophosphate [PF_6_]^−^, bis(trifluoromethylsulfonyl)imide [NTf_2_]^−^) were experimentally determined and subsequently used for PC‐SAFT parameter estimation. The so‐determined parameters were used to predict experimental molecular precursor solubility in ILs and infinitely diluted activity coefficients of various solvents in ILs. The parameters were further compared to modeling results using classical parametrization methods (use of liquid‐density data only for the molecular PC‐SAFT and the ion‐based electrolyte PC‐SAFT). As a result, the modeled precursor solubilities using the new approach are much more precise than using the classical parametrization methods, and required binary parameters were found to be much smaller (if needed). In sum, including the pure‐component vapor‐pressure data of ILs opens the door towards parameter estimation that is not biased by mixture data. This procedure might be suitable also for polymers and for all kind of ionic species but needs extension to ion‐specific parametrization in the long term.

## Graphical abstract

**The conventional way** to fit ionic liquid parameters of equations of state to density is compared using a new method. The experimental vapor pressures as well as the density have been used as input to the model parameter estimation. The work was performed within the framework of the EOS PC‐SAFT (perturbed‐chain statistical associating fluid theory). The new method allows modeling density and vapor pressure accurately, and still allows for an improved modeling of the solubility of precursors in ionic liquids.

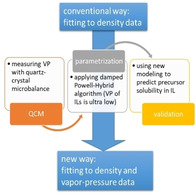

## 1 Introduction

Handling electrolyte chemistry is a complex matter, with various forces induced to the solution and overlapping each other by the electrolytes. Although electrolytes may be beneficial for several applications by tailor‐made influences on phase equilibria through salting‐out or salting‐in effects and phase breaking (e. g., azeotropes), they often negatively influence other system properties, cause corrosion issues or difficulties in technical equipment. Nevertheless, solubility in electrolyte medium is required for any application.

As a special class of electrolytes, ionic liquids (ILs) have gained considerable interest in the research community and in industry. The mutual solubility of the ILs and organic compounds is the main driving force for possible enhancements in industrial applications. On pilot scale, ILs show great potential in synthesis of e. g. metal nanoparticles,[^1^, ^2^] as a substitute solvent to conventional volatile compounds or as additive to tune chemical reactions.^[3]^ In extraction processes,[^4^, ^5^, ^6^] ILs may be custom‐designed as extraction agents, which is fully miscible with the solute but only marginally miscible with the solvent. Even in bioreactions,^[7]^ ILs may be advantageous with designed hydrophobicity for improved reactant solubility.^[8]^ The success behind such operation units rely on improved solubility in the employed IL.

Most research involves imidazolium‐based 1‐alkyl‐3‐methyl‐imidazolum [C_n_mim]‐ILs. These ILs are synthesized in well‐established routines and are widely considered in research as the way‐to‐go ILs. In general, ILs have a remarkably low vapor pressure due to their ionic character while remaining in liquid state below 100 °C or even at room temperature (RTILs) with reduced lattice energy. Additional thermal and chemical stability suggest that ILs are still promising solvents.

A detailed knowledge about interactions in systems with ILs is immanent. The complexity of IL solutions requires thermodynamic models for *a priori* predictions of phase equilibria and precursor solubility in ILs. Therefore, thermodynamic models have been developed with various approaches for the electrolyte forces. Commonly, electrolyte interactions are covered by introducing an additional framework. The electrolyte term is often either based on the Debye‐Hückel theory or the mean spherical approximation (MSA). A thermodynamic model for electrolytes predominantly relies on the model parameters for the ionic species to effectively predict system properties or phase equilibria. The parameter optimization for molecular ILs or their ionic species is a millennial task in electrolyte thermodynamics. Providing generally valid parameters demands experimental solvent‐independent pure‐IL data. State‐of‐the‐art parameter estimation for common electrolytes is usually achieved by using data of aqueous solutions e. g., osmotic coefficients, mean ionic activity coefficients and vapor pressure depressions. Plenty of work was devoted to the parameter estimation of ionic species in aqueous media with various electrolyte models, e. g. electrolyte equations of state (EOS, e. g. eCPA – electrolyte Cubic Plus Association or ePC‐SAFT – electrolyte Perturbed‐Chain Statistical Associating Fluid Theory) or electrolyte G^E^‐models (e. g. eNRTL, COSMO‐RS‐ES). The EOS ePC‐SAFT was implemented^[9]^ and then revised with a new modelling strategy for electrolytes following an ion‐specific approach.^[10]^ eCPA was first developed with salt‐specific parameters^[11]^ and was later extended with ion‐specific parameters.^[12]^ In general, good prediction to quantitative agreement is achieved as long as aqueous systems are described, e. g. mixtures alcohol+water+salt. Reasons for modeling difficulties in the transition from aqueous systems to water‐free solvents with aqueous pure‐component parameters are for example the change in the dielectric decrement with rising electrolyte concentration or varying solvation enthalpies.

A possible solution to regress less biased pure‐component parameters is achieved using experimental data containing solutions with varying solvents. This could potentially reduce the dependency of estimated parameters on one specific solvent. Ultimately, unbiased pure‐component parameters can only be regressed from pure properties. Vapor pressure might be a valid pure‐component property for unbiased parameter estimation, and including vapor pressures is state‐of‐the‐art to parameterize classical organic solvents. The vapor pressure of ILs is very small, which was the main reason until now to not include such data in the pure‐component parameter estimation.

The extremely low vapor pressure of ILs, about 10^−5^ Pa, is an important trade for many industrial applications. In comparison to inorganic salts, the vapor pressure of ILs is measurable at temperature of about 100–200 °C. Accessing the vapor pressure of ILs can thus be a first step towards unbiased parameter estimation.

In the work of Maia et al.^[13]^ two ILs, namely [C_2_mim][NTf_2_] and [C_4_mim][NTf_2_], have been parametrized within CPA including their very low vapor pressures. CPA^[14]^ is a combination of the Soave‐Redlich‐Kwong cubic EOS and the association theory of Wertheim.[^15^, ^16^] Resulting pure‐component parameter sets, including different association schemes, were tested against a variety of thermodynamic properties, e. g. vapor‐liquid equilibria (VLE) with the sour gas CO_2_ or liquid‐liquid equilibria (LLE) with water. For all sets, the predictions are unsatisfying and correlations with binary interaction parameters and solvation energies were accounted for, bringing the ARD% down to about 10 %. To the best of our knowledge, this is the only other workgroup to included vapor pressure of ILs in the parameter estimation.

ILs have already been parametrized with ePC‐SAFT based on liquid density in previous publications.[^4^, ^17^, ^18^] Therefore, ion‐specific parameters have been regressed including an IL‐cation chain length dependency with a total 16 IL‐anions and 8 IL‐cation groups investigated. Using these pure‐component parameters, gas solubility^[19]^ and liquid‐liquid equilibria with water^[5]^ and other solvents^[5]^ have been modelled successfully. 1‐Decyl‐3‐methyl‐imidazolium [C_10_mim]‐ILs, parametrized to liquid density in a molecular approach, have been applied to model LLE of binary and ternary systems with water and 1‐butanol with PC‐SAFT.[^20^, ^21^] PC‐SAFT was also used for newly developed thiophene‐based THT‐ILs.^[22]^ The obtained pure‐component parameters have then been used to calculate the solubility of the ILs in water at 298 K. As pure components, ILs are dissociated only to a certain extent,^[23]^ and the dissociation depends on the medium conditions. In the present work, ILs were considered as a molecule rather than as ionic species. This decision was made to test the influence of the parameterization strategy on modeling results of the precursor solubility in ILs.

In this work, the vapor pressure and liquid density of 16 1‐ethyl‐3‐methyl‐imidazolum [C_2_mim]‐ILs with various IL‐anions, varying from small to very bulky and common to uncommon molecules, were used to regress IL‐specific PC‐SAFT pure‐component parameters. The regressed parameters were tested against thermodynamic solubility data in form of VLE, LLE and activity coefficients at infinite dilution (IDAC). The predictions were compared to results with pure‐component parameters based on liquid density only. Additionally, a comparison of the molecular approach to the ionic parameter sets within ePC‐SAFT was drawn.

## Experimental Section

### Parameter Estimation and Thermodynamic Modelling with PC‐SAFT

#### Vapor Pressure Measurements

Vapor pressures of ILs were measured by using the quartz crystal microbalance (QCM) method.^[24]^ An IL sample was placed in an open cavity (Langmuir evaporation) inside the thermostatted block and exposed to a vacuum (10^−5^ Pa) with the entire open surface of the loaded in the cavity compound. The QCM‐sensor was mounted directly above the measuring cavity with the sample. During the evaporation in a high vacuum, a certain amount of the sample was condensed on the quartz crystal surface. The change of the vibrational frequency of the quartz crystal was recorded. It is related to the mass of the IL Δm deposited on the crystal according to the Sauerbrey equation^[25]^:

(1) $$\Delta f=-C\;\cdot \;f{}^{2}\;\cdot \;\Delta m\;\cdot \;S{}_{C}{}^{-1}$$

where *S*_C_ is the surface of the crystal, C is a constant, *f*=6 MHz. Eq. 1 is applicable only for Δ*f*≪*f*. Therefore, the maximal Δ*f* didn't exceed 2 kHz. As Δ*f* is proportional to the mass uptake rate and to the corresponding vaporization rate d*m*_vap_/d*t* of the low volatile sample under study We can re‐write Eq. 1 in simplified form:

(2) $$dm{}_{\mathrm{vap}}/dt=K\;df/dt$$

The experimental constant K comprises the parameters of the quartz crystal, the thermophysical (density and viscosity) properties of the deposited IL sample, the configuration of the vacuum chamber and the distance between sample and QCM. An possible influence from temperature variation on the thermophysical properties of the deposited on the sensor IL was studied with the help of the thermally very stable ionic liquid [C_10_mim][NTf_2_]. The study was performed at four temperatures of the quartz crystal between 303 and 343 K. These different conditions were able significantly change the density and the viscosity of the material deposited on the QCM sensor and, correspondingly, the recorded signal. From the obtained experimental results, the density and viscosity changes in the significantly different experimental conditions have no systematic influence (non‐systematic deviations are lower than 10 %) on the signal recorded with the QCM sensor. This finding has allowed to set the *K*‐value from eq 2 as a robust constant for the arrangement of our experimental setup in the broad range of temperatures maintained on the QCM‐sensor. This finding was also important to convert the df/dt‐values directly measured with the QCM to the vapor pressure p. Indeed, a combination of eq 8 with the Knudsen equation leads to the calculation of the vapor pressure p:

(3) $$p=\frac{Kdf/dt}{\alpha SK_{C}}\sqrt{\frac{2\pi RT}{M}},$$

where *α* is the condensation coefficient; *S* is the surface of the sample; *K*_C_ is the Clausing factor for the cavity; *R*=8.314462 J ⋅ K^−1^ ⋅ mol^−1^; *T* is the temperature of the sample in K; *M* is the molar mass of species in the vapor phase. Eq 3 can be rearranged in the following way:

(4) $$p=\frac{K}{\alpha SK_{C}}\sqrt{2\pi R}\;\frac{df}{dt}\sqrt{\frac{T}{M}}=K{}^{'}\frac{df}{dt}\sqrt{\frac{T}{M}}$$

where *K*' encompasses now all constants presented in the Knudsen and in the Sauerbrey equations, as well as the configuration of the experimental setup. The *K*'‐value is specific for the experimental setup used in this study and it was determined from the QCM experiments with a series of ionic liquids [C_n_mim][NTf_2_], [C_n_Py][NTf_2_], and [C_n_C_n_im][NTf_2_] where reliable data on absolute vapor pressures and vaporization enthalpies were available.[^26^, ^27^, ^28^, ^29^, ^30^, ^31^] No obvious dependence of *K*'‐values on the type or symmetry of the cation, as well as on the chain length of the alkyl substituent was detected. Thus, an average value *K*'=(9.5±1.1) ⋅ 10‐6 Pa ⋅ s⋅kg^1/2^ ⋅ Hz^−1^ ⋅ K^−1/2^ ⋅ mol^−1/2^ was calculated and used to convert the experimental rates of the frequency change into the vapor pressure values. In order to detect and avoid any possible effect of impurities on the measured mass loss rate, a typical experiment was performed in a few consequent series with increasing and decreasing temperature steps. Every series consisted of 7 to 11 temperature points of mass loss rate determination. Several runs have been performed to test the reproducibility of the results. The primary experimental results of the QCM studies are given in the ESI (Table S1). The absence of decomposition of IL under experimental conditions was controlled using spectroscopy. The residual amount of IL in the cavity, as well as the IL‐deposit on QCM were analyzed by ATR‐IR spectroscopy. No changes in the spectra have been detected.

#### Parameter Estimation at Very Low Vapor Pressure

The estimation of pure‐component parameters consists of two cycles. An inner cycle for the iteration of the thermodynamic properties and a superordinate cycle for the iteration of the pure‐component parameters. Commonly, the parameter estimation in both cycles utilizes a standard Levenberg‐Marquardt algorithm. Extremely low vapor pressures result in a numerically challenging task with objective functions very close to zero that lead to unstable and diverging iteration steps. The iteration step in this case is greater than the distance to the actual null. Additionally, for the superordinate cycle for parameter estimation, the Levenberg‐Marquardt algorithm frequently gets stuck in local minima. That said, the original iteration methods are not suitable for the incorporation of very low vapor pressures of ILs for parameter estimation. Both cycles were improved in this work. For the superordinate cycle, the procedure was changed to a genetic algorithm, also to overcome the local minima problems. The inner cycle for the vapor‐pressure iteration was altered to a damped Powell‐Hybrid algorithm.

The iteration in the fitting routine of the genetic algorithm is divided into selection, recombination and mutation of a random starting population. The starting population comprises $n_{I}$ individuals with the aim to enhance the fitness of the individuals throughout the iteration steps. Here, the individuals comprise a set of accompanying pure‐component parameters, transformed into binary data. The fitness F (Equation (5)) was calculated via the secondary cycle for the thermodynamic properties, where $y_{j}^{calc}$ and $y_{j}^{exp}$ are calculated and experimental vapor pressure and liquid density of pure IL, respectively.

(5) $$F\left(x_{I}\right)={\left[\sum_{j=1}^{n}{\left(y_{j}^{calc}-y_{j}^{exp}\right)}^{2}\right]}^{-1}$$

In the selective step the individuals are in competition. The fitness of a random set of individuals was compared and the individual with the higher fitness is selected for the next procedure step. In the third step, the selected individuals were recombined. The mechanism cuts two individuals and recombines the endings to the counterpart.

The Powell‐Hybrid algorithm, used in the secondary cycle, is a method often used to find solutions to non‐linear least square problems. It is known to show surpassing convergence for multi‐dimensional optimizations. Similar to the commonly used Levenberg‐Marquardt method for parameter estimation within PC‐SAFT, the Powell algorithm uses the Gauss‐Newton method but combined with a “dog leg” step. First, the steepest direction is determined. It is the negative transposed Jacobian multiplied by the function to be minimized (Eq. 6).

(6) $${-g}_{steapest}=-J{\left(x\right)}^{T}\cdot f\left(x\right)$$

Thereafter, the step size is chosen. It is supposed to stay in a trusted region of a specific radius r near the ordinate of the predecessor function value $x_{n}$ . In this work and to controle the stability of the iteration, the stepsize (i. e., radius *r*) was allowed to be in the order of magnitude of the previous step, monitored by a gain ratio. Therewith, the method may initially require more iteration steps, but the chance of diverging steps is drastically reduced.

The parameter regression overall followed the minimization of the objective function (Equation (7)), including experimental and modelled liquid density ($\rho_{i}^{exp\;}$ and $\rho_{i}^{calc\;}$ , respectively) and vapor pressure ($p_{i}^{LV,exp}$ and $p_{i}^{LV,calc}$ ). *NP* is the number of data points available for the experimental property.

(7) $$OF=min!=\frac{1}{NP}\cdot \sum_{i}^{NP}\left|\frac{\rho_{i}^{exp}-\rho_{i}^{calc}}{\rho_{i}^{exp}}\right|+\sum_{i}^{NP}\left|\frac{p_{i}^{LV,exp}-p_{i}^{LV,calc}}{p_{i}^{LV,exp}}\right|$$

Including the vapor pressure into the parameter estimation was tested and compared to the original procedure only accounting for liquid density and ePC‐SAFT results. The results were quantified by comparing to experimental data by using absolute average deviation (AAD) and average relative deviation (ARD%) (Equation s (8) and (9)) between PC‐SAFT and the experimental data.

(8) $$AAD=\frac{1}{NP}\cdot \sum_{i}^{NP}\left|y_{i}^{calc}-y_{i}^{exp}\right|$$

(9) $$ARD\%=\frac{100}{NP}\cdot \sum_{i}^{NP}\left|1-\frac{y_{i}^{calc}}{y_{i}^{exp}}\right|$$

For the assessment of the LLE, AAD values are given mole‐based [mol/mol] for the IL solubility at constant temperature. For VLE, all AAD‐values are given for pressure difference in [bar] at certain concentration of the IL. For the evaluation of the pure IL vapor pressure after parameter estimation, AAD is given in [10^5^ Pa]. By convention, IDAC are dimensionless, so are the respective AAD.

#### Thermodynamic modelling with PC‐SAFT

The thermodynamic model PC‐SAFT was applied for the prediction of phase equilibria based on the new parameter‐estimation strategy for the ILs. Phase equilibria of systems containing [C_2_mim]‐ILs and various solvents and/or gases are predicted utilizing the isofugacity criterion as depicted in Eq. 10.

(10) $$\varphi_{i}^{I}\cdot x_{i}^{I}=\varphi_{i}^{II}\cdot x_{i}^{II}$$

The phase equilibrium is reached when the chemical potential of each component *i* are equal in each phase (*I* and *II*) at constant pressure and temperature. The PC‐SAFT equation of state and the calculation of the fugacity coefficient is derived from the residual Helmholtz energy $a^{res}$ . For detailed information, the reader is directed to the original publication.^[32]^ $a^{res}$ is the summation of three independent terms (Equation 11).

(11) $$a^{res}=a^{hard\;chain}+a^{dispersion}+a^{association}$$

Interactions are considered caused by the repulsion of the molecules (hard chain), unspecific attractive forces (dispersion) as well as for hydrogen‐bonding interactions (association). The single independent terms require pure‐component parameters. The [C_2_mim]‐ILs were modeled as associating compounds. The total number of pure‐IL parameters is thus five: the number of segments $m_{i}^{seg}$ , the segment diameter $\sigma_{i}$ , the dispersion‐energy parameter $u_{i}/k_{B}$ and the association‐energy parameter $\epsilon^{AiBi}/k_{B}$ as well as the association‐volume parameter $\kappa^{AiBi}$ . Here, $k_{B}$ is the Boltzmann constant. Non‐associating compounds (in this work: alkanes and sour gases) only use the first three parameters.

By differentiation, the fugacity coefficient is calculated (Eq. 12).

(12) $$\ln\left(\varphi_{i}\right)=\frac{\mu_{i}^{res}}{k_{B}\cdot T}-\ln\left(1+\left(\frac{\partial \left(\frac{a^{res}}{k_{B}\cdot T}\right)}{\partial \rho }\right)\right)$$

Activity coefficients at infinite dilution (IDAC) of the component i in IL can be calculated from the fugacity coefficients in the mixture $\varphi_{i}$ divided by the fugacity coefficient at infinite dilution of component i in the IL $\varphi_{i}^{\infty ,IL}$ (Eq. 13)

(13) $$\gamma_{i}^{\infty ,IL}=\frac{\varphi_{i}\left(T,p,\vec{x}\right)}{\varphi_{i}^{\infty ,IL}\left(T,p,x_{i}\to 0\right)}$$

IDAC can be transformed into solubility of compound *i* in the IL $x_{i}$ with Equation (14). This work thus focuses on solubility of gaseous and liquid non‐ionic compounds or precursors in IL.

(14) $$x_{i}=1/\gamma_{i}^{\infty ,IL}$$

Berthelot‐Lorentz combining rules for the segment diameter and dispersion energy were applied for mixtures for component i and component j (Eq. (15) and 16).

(15) $$\sigma_{ij}=\frac{1}{2}\left(\sigma_{i}+\sigma_{j}\right)$$

(16) $$u_{ij}=\sqrt{u_{i}u_{j}}\left(1-k_{ij}\right)$$

For associating compounds, the mixing rules of Wolbach and Sandler for association energy and association volume were applied according to Equation (17) and 18.

(17) $$\epsilon^{AiBj}=\frac{1}{2}\left(\epsilon^{AiBi}+\epsilon^{AjBj}\right)$$

(18) $$\kappa^{AiBj}=\sqrt{\kappa^{AiBi}\cdot \kappa^{AjBj}}{\left(\frac{\sqrt{\sigma_{i}\sigma_{j}}}{\frac{1}{2}\left(\sigma_{i}+\sigma_{j}\right)}\right)}^{3}$$

Equation (16) also introduces the binary interaction parameter $k_{ij}$ used as a degree of freedom to alter the dispersion energy in the mixture and thus correlate the system. For comparison, the prediction of solubility data (phase equilibria and $\gamma_{i}^{\infty ,IL}$ ) with the classical parameter estimation and the new parameter estimation (omitting and including vapor‐pressure data) is of great interest in this work. Therefore, for a first study $k_{ij}$ will not be used and set to zero in the first place. Only in this case an unprejudiced discussion is possible. Still, modelling the experimental data correctly is important for further studies of more complex systems. Hence, correlations again for both parameter sets were performed. The binary interaction parameter $k_{ij}$ is fitted to represent the experimental data with lowest mean square deviation from the experimental data.

## 2 Results and Discussion

### 2.1 Extremely Low Vapor Pressures of ILs

The considered ILs consisted of one of the 16 IL‐anions, including the most common IL‐anions, like [NTf_2_]^−^ or [BF_4_]^−^ as well as bulkier ones (e. g. [(C_2_H_5_O)_2_PO_2_]^−^). An overview is given in Table 1.

**Table 1:** IL‐anions considered in this work.

IL‐anion	Formula	Structure
Trifluoroacetate	[CF_3_CO_2_]^−^	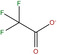
Bis(trifluoromethyl‐sulfonyl)imide	[NTf_2_]	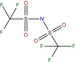
Hexafluorophosphate	[PF_6_]	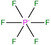
Trifluoromethanesulfonate	[CF_3_SO_3_]^−^	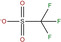
Methanesulfonate	[CH_3_SO_3_]^−^	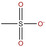
Tetrafluoroborate	[BF_4_]^−^	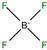
Tetracyanoborate	[B(CN)_4_]^−^	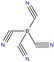
Thiocyanate	[SCN]^−^	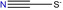
Tris(pentafluoroethyl) trifluorophosphate	[(C_2_F_5_)_3_PF_3_]^−^	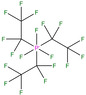
Diethyl phosphate	[(C_2_H_5_O)_2_PO_2_]^−^	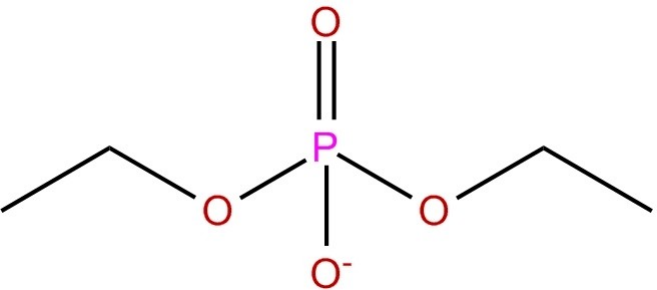
4‐methylbenzenesulfonate	[4‐CH_3_−Ph−SO_3_]‐	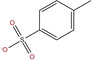
Tricyanomethanide	[C(CN)_3_]^−^	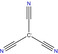

A summary of experimental vapor pressures in the magnitude of 10^−5^ Pa and the experimental temperature ranges for the series of [C_2_mim]‐ILs determined in this work is given in Table 2. The preliminary data on the temperature dependency of the frequency shift velocities were reported earlier.^[33]^ For this work, the experimental vapor pressures have been derived from these data and reported for the first time. Available in the literature data for [C_2_mim][NTf_2_][^27^, ^28^] were used for the calibration of the sensor. Detailed experimental information required to determine the extremely low vapor pressure, including the temperature dependence of the frequency shift rate and the enthalpies of vaporization, is given in the ESI (Table S1).

**Table 2:** Overview of the experimental vapor pressure of the investigated ILs and number of data points NP.

Ionic Liquid	T range/K	Vapor Pressure/10^−5^ Pa	NP
[C_2_mim][NTf_2_]	362–395	0.4–10.3	14
[C_2_mim][SCN]	392–435	0.29–20.2	18
[C_2_mim][CF_3_CO_2_]	361–405	0.37–27.9	17
[C_2_mim][CF_3_SO_3_]	395–432	0.76–21.1	16
[C_2_mim][(C_2_H_5_O)_2_PO_2_]	375–412	0.49–26.0	16
[C_2_mim][PF_6_]	414–457	0.65–22.4	18
[C_2_mim][BF_4_]	412–454	1.0–28.4	16
[C_2_mim][B(CN)_4_]	380–428	0.23–18.1	30
[C_2_mim][C(CN)_3_]	400–445	0.35–15.9	20
[C_2_mim][CH_3_SO_3_]	402–445	0.75–34.0	28
[C_2_mim][(C_2_F_5_)_3_PF_3_]	350–395	0.1–10.1	20
[C_2_mim][4‐CH_3_−Ph−SO_3_]	440–482	0.72–26.9	18

### 2.2 Pure Component Parameters

Using a molecular approach to describe ILs within PC‐SAFT requires incorporation of association. All considered [C_2_mim]‐ILs were modelled with the same association scheme (2B).^[34]^ The selected association scheme was found to give more reasonable results for the pure‐component parameters and related results in binary systems compared to schemes with more association sites. Still, this is a limiting assumption that is strongly dependent on the IL‐anion type. The 2B scheme might be dropped for very complex molecules with multiple association sites. Liquid density is the second experimental input data to parameter estimation of the new approach and the basis for the original method. Liquid density data can be measured with less experimental effort than vapor pressure; such data is readily available in the literature. Density data in the temperature range 283–457 K at ambient pressure used in this work is summarized in Table 3.

**Table 3:** Overview of the experimental liquid density of the investigated ILs at ambient pressure.

Ionic Liquid	T range/K	NP	Reference
[C_2_mim][NTf_2_]	362–395	14	[35]
[C_2_mim][SCN]	392–439	18	[36]
[C_2_mim][CF_3_CO_2_]	361–405	17	[37]
[C_2_mim][CF_3_SO_3_]	394–432	16	[38]
[C_2_mim][(C_2_H_5_O)_2_PO_2_]	375–412	16	[39]
[C_2_mim][PF_6_]	414–457	18	[40]
[C_2_mim][BF_4_]	412–454	16	[41]
[C_2_mim][B(CN)_4_]	380–428	30	[42]
[C_2_mim][C(CN)_3_]	400–447	20	[43]
[C_2_mim][CH_3_SO_3_]	402–445	28	[44]
[C_2_mim][(C_2_F_5_)_3_PF_3_]	283–338	12	[45]
[C_2_mim][4‐CH_3_−Ph−SO_3_]	303–363	13	[44]

**Figure 1:**
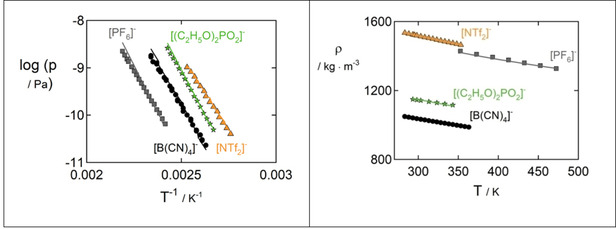
Left: Logarithmic vapor pressure in Pa of a selection of four [C_2_mim]‐ILs with varying IL‐anions over inverse temperature. Right: Atmospheric liquid density of a selection of [C_2_mim]‐ILs with varying IL‐anions over temperature. Symbols are experimental data: squares: [PF_6_]^−^, circles: [B(CN)_4_]^−^, stars: [(C_2_H_5_O)_2_PO_2_]^−^, triangles [NTf_2_]^−^. Lines are results obtained with PC‐SAFT pure‐component parameters (c.f. Table 4).

Figure 1a exemplarily shows results of the pure‐component vapor pressures modeled with PC‐SAFT and the parameters listed in Table 4. The parameter estimation with the newly developed numerical code was successful for all investigated ILs. For simplicity, only four ILs are shown. Figure 1b presents the results of PC‐SAFT modeled liquid density for the respective ILs. Both results shown in Figure 1a and Figure 1b have been obtained simultaneously. The pure‐component parameters obtained from the parameter estimation with and without the use of experimental vapor‐pressure data are listed in Table 4 and Table 5. With the new methods for parametrization, the results for the vapor pressure and liquid density are in very good agreement with the experimental data. This indicates that the new approach is a valid opportunity providing the advantage of accessing the extremely low vapor pressure. PC‐SAFT is well‐known for modeling polymers.^[46]^ Similar to ILs, polymers exhibit very low vapor pressures. Thus, this work might also contribute to enhanced parametrization for polymers.

**Table 4:** Pure‐component parameters for the [C_2_mim]‐ILs with various IL‐anion derived from regression to vapor pressure and liquid density. AAD given for the vapor pressure (VLE) and ARD% for the liquid density (pVT). All ILs were modeled with a 2B association scheme.

Ionic Liquid	$m_{i}^{seg}$	$\sigma_{i}$ /Å	$u_{i}$ /K	$\epsilon^{AiBi}/k_{B}$ /K	$\kappa^{AiBi}$	AAD ⋅ 10^−6^ VLE/Pa	ARD% pVT
[C_2_mim][NTf_2_]	6.5240	3.9733	342.0918	4016.5728	0.1100	4.1115	3.1637
[C_2_mim][SCN]	2.6977	4.5778	819.4725	1586.2271	0.0385	0.2242	0.9394
[C_2_mim][CF_3_CO_2_]	2.6472	4.7956	710.1802	2934.0415	0.0286	5.1603	3.6787
[C_2_mim][CF_3_SO_3_]	3.4288	4.5467	684.3209	2765.8608	0.0028	11.1875	1.1728
[C_2_mim][(C_2_H_5_O)_2_PO_2_]	6.5017	3.6800	241.7846	9801.0256	0.0033	19.4318	6.2123
[C_2_mim][PF_6_]	3.5154	4.3956	718.9626	2140.5128	0.0043	0.9122	2.4003
[C_2_mim][BF_4_]	2.7238	4.5956	840.4528	1903.6386	0.0166	48.8369	2.1593
[C_2_mim][B(CN)_4_]	2.9062	5.0400	767.5099	1202.4908	0.0416	0.3557	6.1372
[C_2_mim][C(CN)_3_]	3.0574	4.7156	765.3143	3334.3590	0.0012	2.6219	2.0024
[C_2_mim][CH_3_SO_3_]	2.8749	4.5378	546.2418	6887.4725	0.0395	53.7122	0.0514
[C_2_mim][(C_2_F_5_)_3_PF_3_]	3.6251	5.2844	546.7297	3372.2589	0.0237	0.6519	0.0188
[C_2_mim][4‐CH_3_−Ph−SO_3_]	2.4845	5.2444	460.6132	9981.0501	0.0731	0.9771	0.7725

**Table 5:** Pure‐component parameters for the [C_2_mim]‐ILs with various IL‐anion derived from regression to liquid density only. ARD% given for the liquid density (pVT). All ILs were modeled with a 2B association scheme.

Ionic Liquid	$m_{i}^{seg}$	$\sigma_{i}$ /Å	$u_{i}$ /K	$\epsilon^{AiBi}/k_{B}$ /K	$\kappa^{AiBi}\;$	ARD% pVT
[C_2_mim][NTf_2_]	5.3290	4.1378	293.7473	4997.2161	0.0994	0.0075
[C_2_mim][SCN]	1.8293	5.0909	715.8919	1465.5609	0.0010	0.0587
[C_2_mim][CF_3_CO_2_]	2.1606	5.0221	571.6428	986.8769	0.0424	0.0164
[C_2_mim][CF_3_SO_3_]	2.4568	4.8920	494.6771	300.0000	0.0151	0.0353
[C_2_mim][(C_2_H_5_O)_2_PO_2_]	2.4446	5.3165	542.6473	1708.2565	0.0582	0.0087
[C_2_mim][PF_6_]	2.7759	4.5109	312.9214	3493.9923	0.0757	0.0600
[C_2_mim][BF_4_]	3.1489	4.2974	536.8010	8986.5509	0.0600	0.0184
[C_2_mim][B(CN)_4_]	2.4791	5.0969	418.4029	1027.1867	0.0629	0.0226
[C_2_mim][C(CN)_3_]	1.9306	5.2859	536.7422	436.8861	0.0812	0.0096
[C_2_mim][CH_3_SO_3_]	1.8116	5.1790	423.1505	3992.9139	0.0471	0.0322
[C_2_mim][(C_2_F_5_)_3_PF_3_]	3.0462	5.4812	417.7560	3397.6966	0.0010	0.0001
[C_2_mim][4‐CH_3_−Ph−SO_3_]	2.8898	5.1569	807.3601	2030.4989	0.0497	0.0195

The integration of experimental vapor‐pressure data into the parameter estimation slightly decreases the accuracy in the prediction of the liquid density. This is a mathematically necessary and consistent result. The ARD for the density of the 16 ILs increased from 0.24 % (Table 5) to 2.39 % (Table 4) upon also incorporating the vapor‐pressure data into the parameter estimation. Compared to other substances modeled with PC‐SAFT so far, this is still a very god result. With the pure‐component parameters obtained from density only, the vapor pressure is largely overestimated. In the new parametrization method, ILs are modeled as longer and thinner molecules. Hence, both, increasing the segment number and decreasing the segment diameter help reducing the vapor pressure to very low values. Similarly, the dispersion energy is increased. The already high values for the dispersion energy represent the existing unidirectional forces in the IL that are also reason for the liquid state around room temperature. Most ILs only slightly change in the values for the pure‐component parameters when including the vapor pressure. Still, sometimes the parameters drastically change to meet the experimental data (c.f. [C_2_mim][(C_2_H_5_O)_2_PO_2_]). Only including liquid densities in the parameter estimation gives a potentially large number of possible compositions that will represent the experimental data well. The incorporation of low vapor pressure data reduces the degrees of freedom, which possibly results in a larger difference for the obtained parameter sets.

### 2.3 Comparison of the parametrization methods by prediction and correlation of phase equilibria

Equilibrium predictions and correlations have been performed with all literature data on solubility available during the research. The VLE of sour gases (H_2_S and CO_2_) with ILs was a main source for experimental data. Literature on IDAC of various volatile compounds, e. g. alcohols and alkanes, was also extensively available. Only in the case of [C_2_mim][NTf_2_] a liquid‐liquid equilibrium (LLE) could be investigated. A list of pure‐component PC‐SAFT parameters for the organic compounds used in this work is given in Table 6. A summary on investigated systems and the respective ARD% and AAD for the two parameter sets presented in Table 4 (VLE+pVT data used for the parameter estimation) and in Table 5 (only pVT data used for the parameter estimation) is listed in Table 7.

**Table 6:** Pure‐component parameters for the organic compounds used in the prediction and correlation of phase equilibria with [C_2_mim]‐ILs.

Organic Compound	$m_{i}^{seg}$	$\sigma_{i}$ /Å	$u_{i}$ /K	$\epsilon^{AiBi}/k_{B}$ /K	$\kappa^{AiBi}\;$	Association Scheme	Reference
Water	1.2047	2.7927	353.94	2425.7	0.0451	2B	[47]
CO_2_	2.0729	2.7852	169.21	–	–	–	[32]
H_2_S	1.6941	3.0214	226.79	–	–	–	[48]
Methanol	1.5255	3.2300	188.9	2899.5	0.0352	2B	[49]
Ethanol	3.1752	2.8283	170.287	2502.21	0.0324	2B	[49]
1‐Propanol	3.2652	3.1474	225.163	2151.08	0.0153	2B	[49]
2‐Propanol	3.0929	3.2085	208.42	2253.9	0.0247	2B	[49]
1‐Butanol	4.2102	3.0741	219.92	1890.72	0.0067	2B	[49]
Benzene	2.4653	3.6478	287.35	–	–	–	[32]
Pentane	2.6896	3.7729	231.2	–	–	–	[32]
Hexane	3.0576	3.7983	236.77	–	–	–	[32]

**Table 7:** Overview of investigated thermodynamic properties of systems containing [C_2_mim]‐ILs and organic compounds. ARD% and AAD for the predications are given for the two parameter sets of the [C_2_mim]‐ILs in Table 4 (use of vapor pressure and liquid density) and Table 5 (liquid density only).

Organic Compound	Property	Table 4		Table 5		Reference
		*AAD*	*ARD%*	*AAD*	*ARD%*	
[C_2_mim][NTf_2_]						
Water	LLE	0.412	60.320	0.156	22.858	[50]
CO_2_	VLE	61.70	76.36	56.74	65.60	[51]
[C_2_mim][SCN]						
Water	IDAC	0.64	230.63	0.97	350.31	[52]
CO_2_	VLE	1.61	89.55	1.77	98.26	[53]
[C_2_mim][CF_3_CO_2_]						
Water	IDAC	0.51	337.60	0.85	565.40	[54]
CO_2_	VLE	6.47	74.85	6.99	83.08	[55]
[C_2_mim][CF_3_SO_3_]						
Water	VLE	0.03	28.85	0.02	38.10	[56]
Methanol	IDAC	2.64	378.61	3.24	465.63	[57]
[C_2_mim][(C_2_H_5_O)_2_PO_2_]						
CO_2_	VLE	39.60	94.79	36.81	89.11	[58]
Hexane	IDAC	9.92	15.30	67.36	99.49	[59]
Pentane	IDAC	19.50	47.47	40.82	99.02	[59]
[C_2_mim][PF_6_]						
CO_2_	VLE	333.22	83.92	329.57	81.17	[60]
H_2_S	VLE	1.16	10.83	2.08	20.84	[61]
[C_2_mim][BF_4_]						
Water	VLE	0.12	28.75	0.05	20.21	[62]
Water	IDAC	0.42	93.06	0.48	105.80	[63]
Benzene	VLE	0.01	19.77	0.01	29.53	[64]
[C_2_mim][B(CN)_4_]						
Water	IDAC	0.44	25.38	0.54	27.12	[65]
CO_2_	VLE	27.16	82.66	25.43	76.22	[66]
[C_2_mim][C(CN)_3_]						
Water	IDAC	0.21	23.67	0.53	58.95	[67]
CO_2_	VLE	2.32	69.94	2.91	89.95	[53,68]
[C_2_mim][CH_3_SO_3_]						
Water	VLE	0.004	109.09	0.01	221.02	[62]
Water	IDAC	0.89	1138.09	1.13	1451.44	[69]
CO_2_	VLE	27.75	87.02	28.91	91.03	[58]
[C_2_mim][(C_2_F_5_)_3_PF_3_]						
Water	IDAC	4.04	76.09	3.62	68.19	[70]
CO_2_	VLE	8.89	83.36	8.73	82.42	[71]
[C_2_mim][4‐CH_3_−Ph−SO_3_]						
Methanol	IDAC	0.19	72.35	0.96	369.56	[72]
Ethanol	IDAC	0.30	60.18	0.30	60.08	[72]
1‐Propanol	IDAC	0.55	81.34	0.63	93.13	[72]
2‐Propanol	IDAC	0.74	87.27	0.80	94.08	[72]
1‐Butanol	IDAC	0.75	76.40	0.75	76.95	[72]

**Figure 2:**
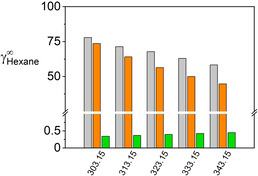
IDAC for the organic compound hexane in [C_2_mim][(C_2_H_5_O)_2_PO_2_]. Gray bars are experimental data, orange bars are predictions performed including vapor pressure in parametrization (Table 4) and green bars are predictions omitting vapor pressure (Table 5). $k_{ij}$ set to zero.

With the exception of the IL [C_2_mim][(C_2_F_5_)_3_PF_3_], the incorporation of vapor pressure into the regression routine for pure‐component parameters results in a more precise prediction of experimental VLE, LLE, and IDAC data. For [C_2_mim][(C_2_F_5_)_3_PF_3_], the results achieved with both parameter sets are similar, with a difference in ARD%‐values of less than 10 %. For all other mixture, the results are in favor of the new parametrization method including vapor pressure. Figure 2 exemplarily depicts the IDAC for hexane in the IL [C_2_mim][(C_2_H5O)_2_PO_2_]. The IDAC are almost quantitatively represented, correctly capturing the decrease with rising temperature, yielding ARD% of 15 %. Only relying on liquid density, the IDAC are strongly underestimated and the temperature‐related decrease of the IDAC data cannot be predicted correctly, yielding ARD% of 99 %.

**Figure 3:**
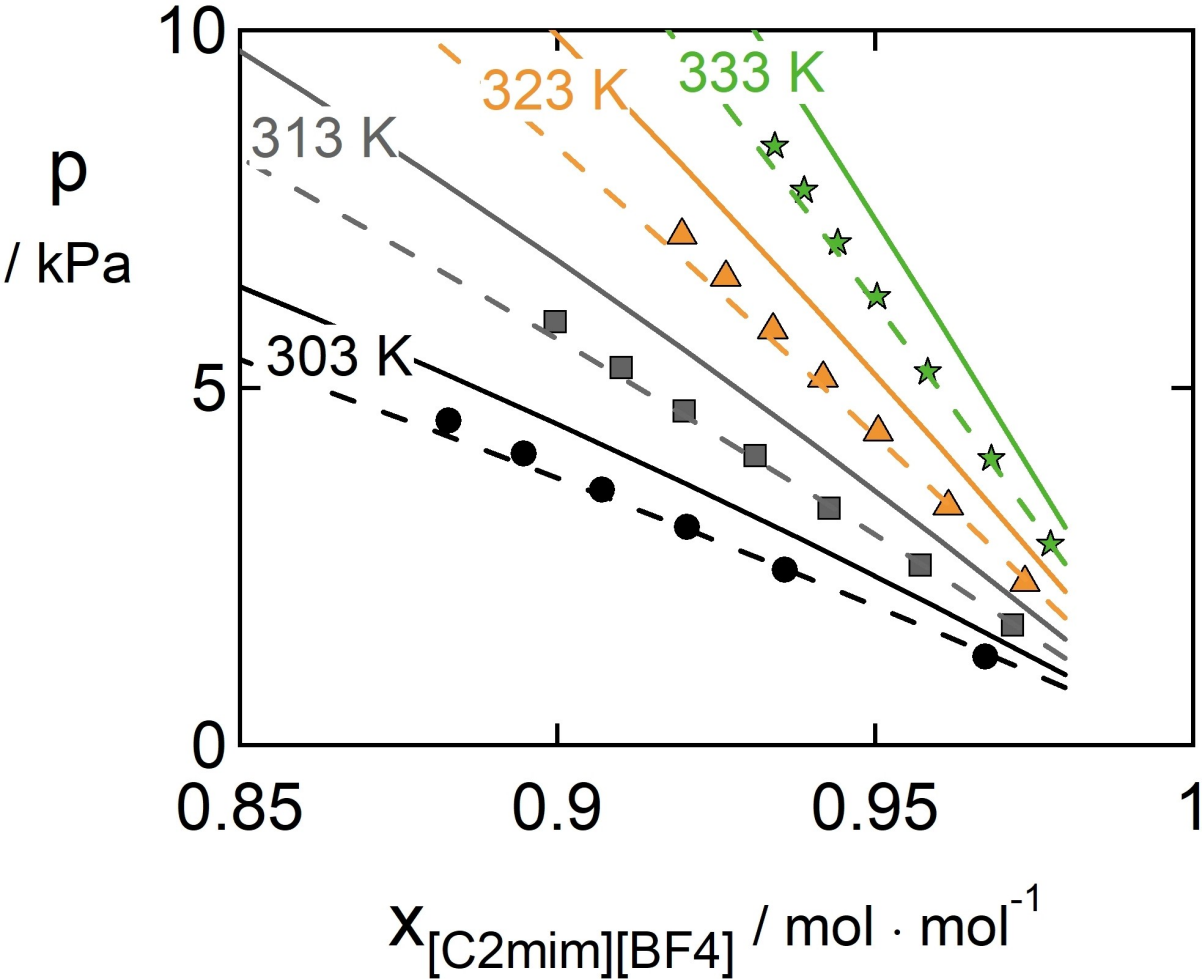
VLE of the system [C_2_mim][BF_4_] – benzene. Symbols are experimental data (circles: 303 K; squares: 313 K; triangles: 323 K; stars: 333 K),^[64]^ solid lines are predictions (pVT+VLE), dashed lines are correlations with $k_{ij}=-0.005$ using the parameters from Table 4 and 6.

Still, the predictions with PC‐SAFT for the investigated systems deviate from the experimental data. For a complete investigation, a satisfying agreement with experimental data is desired. The systems were therefore also correlated with binary interaction parameters $k_{ij}$ between the [C_2_mim]‐ILs and the respective organic compound. For both parameter sets, the correlation was possible and showed good agreement with the experimental data, summarized in Table S2 in the ESI. Using the new parameter estimation method developed in this work (Table 4) allows significantly reducing the magnitude of the binary interaction parameters compared to the classical modeling (Table 5) as well as yielding lower AAD or ARD% values. The correlative ability of PC‐SAFT is depicted in Figure 3 for the VLE of the system [C_2_mim][BF_4_] – benzene at temperatures from 303 to 333 K. The already good predictions (*k_ij_*=0) could be improved by using a very small binary interaction parameter of only −0.005 yielding an ARD% of 2.8 %. For the same system and the original parametrization method, the $k_{ij}$ ‐value was found to be three times higher, still resulting in ARD% of 5.92 (data not shown in the figure).

The LLE of the system water and [C_2_mim][NTf_2_] was well captured in the temperature range of 288 to 318 K by both parameter sets compared to the experimental data (no graphical illustration). The originally better results for the prediction with the parameter set only including liquid density (Table 5) are overcome by better correlation abilities and temperature dependency of the new method (Table 4). The results including liquid density (Table 4) show a more pronounced temperature dependence that would ultimately lead to a critical point at a too low temperature. In contrast, the new method (Table 5) follows the temperature‐dependent solubility data qualitatively. Although giving higher ARD%‐values, this allows a temperature‐independent $k_{ij}$ for the correlation. ARD% are calculated for the solubility of water in the IL as this is by far more important compared to the almost negligible solubility of the IL in water. Just as well, a comparison including the deviation in the IL solubility would impede the analysis as already very small concentrations cause huge deviation in the ARD%‐values. Please also note that more experimental data is available for this system with some scattering in the mutual solubility. For clarity, we decided to only compare results for one data set.^[50]^ This literature is from a series of work devoted to solubility data of various ILs.

**Figure 4:**
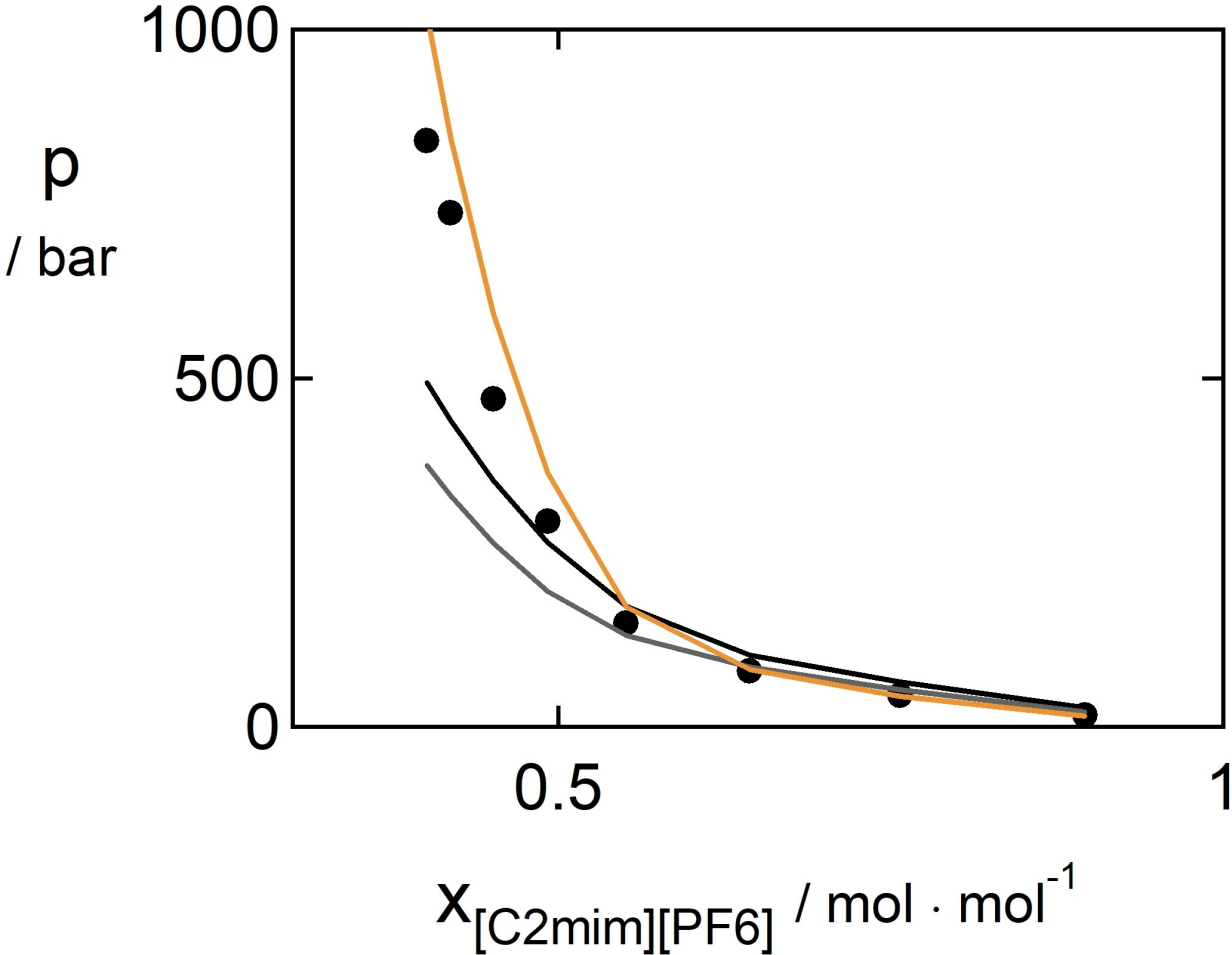
VLE of the system [C_2_mim][PF_6_] – CO_2_ at 343.15 K Comparison of the correlative results from this work (grey line: parameters from Table 4 and 6 and $k_{ij}=-0.088$ ) and black line: parameters from Table 5 and 6 and $k_{ij}=-0.088$ ). ePC‐SAFT prediction (orange line) with ionic parameters according to Ji et al.^[17]^ Circles are experimental data.^[60]^

Usually, it is accepted that binary parameters are required to accurately model phase equilibria. Thus, it was expected that predictions (*k_ij_*=0) cause high deviations compared to experimental data for most of the mixtures. In contrast, the ion‐specific ePC‐SAFT approach was found to be predictive for gas solubility in ILs, with ARD% of usually lower than 10 %. The new parameter set developed in this work (Table 5) was compared to this extremely good ePC‐SAFT result. Indeed, it can be observed in Figure 4 that ePC‐SAFT is superior in modeling the VLE of CO_2_+[C_2_mim][PF_6_].

PC‐SAFT underestimates pressure especially at x_CO2_>0.5, i. e. at high‐pressure conditions (ARD%=31.39 % and 33.70 %, respectively). In contrast the ePC‐SAFT prediction yields lower ARD values of ARD%=14.55. Three reasons might be seen behind this result. First, the cross association for CO_2_ with other associating compounds drastically influences the observed vapor pressure. Cross association was lately found to be the key to quantitative modeling of water+CO_2_ binary systems. The assumption of a 2B association scheme might thus be a reason for the reduced accuracy below x_CO2_>0.5. Second, Ji et al. included high‐pressure data in the parameter estimation while in the present work only atmospheric data were used. Third, an ionic approach for ILs appear suitable. Accounting for Coulomb forces explicitly combined with the new parameter estimation method develop in this work might be the key for a powerful model with most‐universal model parameters. However, this requires a sufficiently large data amount of vapor pressures for ILs with various IL‐anion and IL‐cation pairs; this will then also require mathematical methods decrease the already numerically demanding process.

## 3 Conclusions

This work introduces a new method for parameter estimation for ionic liquids, utilizing their extremely low vapor pressure as experimental input data. Therefore, experimental vapor‐pressure data for a series of [C_2_mim]‐ILs with 16 different IL‐anions have been measured with the QCM method and applied for PC‐SAFT pure‐component estimation. The pure‐component parameters for PC‐SAFT derived from vapor pressure and liquid density were estimated for the ILs using a molecular approach. The new parametrization method has been tested against phase equilibria of systems containing [C_2_mim]‐ILs. For comparison, results with the original parametrization method including liquid density only have been included. In total, 15 VLE, 2 LLE and 15 IDAC were predicted with PC‐SAFT. The utilization of the vapor pressure in the parameter estimation yields a significant improvement in predicting the solubility in ILs (IDAC, VLE, and LLE for correlations and temperature dependence) compared to the classical PC‐SAFT modeling of ILs that uses density data only to estimate the IL parameters. Predictions were compared in terms of ARD% and AAD values. Based on this, the new parametrization method required overall smaller $k_{ij}$ ‐values for the accurate representation of the experimental data. Comparing these results with the ion‐based ePC‐SAFT approach for CO_2_+IL mixtures allows concluding that ePC‐SAFT is especially suitable to predict gas solubility in ILs without binary parameters. A logical next step would thus be to regress ion‐specific pure‐component parameters using the approach developed in this work. In total, this work shows that the pure‐component vapor pressure is vital in the progress towards generalized parameters for compound with low vapor pressures. Accessing extremely low vapor pressure experimentally and in parameter estimation could also be applied to other species exhibiting low vapor presser, e. g. polymers or even for conventional electrolytes such as alkali halides.

## Conflict of interest

The authors declare no conflict of interest.

## Supporting information

As a service to our authors and readers, this journal provides supporting information supplied by the authors. Such materials are peer reviewed and may be re‐organized for online delivery, but are not copy‐edited or typeset. Technical support issues arising from supporting information (other than missing files) should be addressed to the authors.

Supplementary
