# Supplementary material for: Extremely Low Vapor‐Pressure Data as Access to PC‐SAFT Parameter Estimation for Ionic Liquids and Modeling of Precursor Solubility in Ionic Liquids
Source: ChemistryOpen. 2021 Jan 25;10(2):216–26. doi: 10.1002/open.202000258 (PMC7874510; doi:10.1002/open.202000258)
Supplement: Supplementary file 1 — Supplementary [file OPEN-10-216-s001.pdf]

ChemistryOpen

Supporting Information

Extremely Low Vapor-Pressure Data as Access to PC-SAFT Parameter Estimation for Ionic Liquids and Modeling of Precursor Solubility in Ionic Liquids

Mark Bülow, Moritz Greive, Dzmitry H. Zaitsau, Sergey P. Verevkin, and Christoph Held*

Table S1 The results of the temperature dependences of the frequency shift velocities df/dt and absolute vapor pressures p^*_{sat} measured by the QCM for [$C_n$mim][Anion] and vaporization enthalpies $\Delta_l^g H_m^o(T)$ derived from these data.[a]

Run	T [K]	df/dt [Hz·s^{-1}][b]	$10^6 \cdot p^*_{sat}$ [Pa][c]	T^{-1} [K^{-1}]	$R \cdot \ln(p^*_{sat}/p^o)$	$\Delta_l^g H_m^o(T)$ [kJ·mol^{-1}]
[C_2mim][CF_3CO_2]						
$\ln(p^*_{sat}/p^o) = -\frac{69084}{RT_0} - \frac{120594}{R}\left(\frac{1}{T} - \frac{1}{T_0}\right) - \frac{67}{R}\left(\frac{T_0}{T} - 1 - \ln\left(\frac{T}{T_0}\right)\right)$, T_0 = 383.9 K						
1	402.38	0.5565	224	0.002485	-165.6	119.4
	397.01	0.3460	138	0.002519	-169.6	119.7
	391.99	0.2175	86	0.002551	-173.5	120.1
	387.05	0.1368	54	0.002584	-177.4	120.4
	382.08	0.08485	33	0.002617	-181.5	120.7
	377.08	0.05128	20	0.002652	-185.7	121.1
	372.13	0.03085	12	0.002687	-190.0	121.4
	366.93	0.01789	6.9	0.002725	-194.6	121.7
	361.15	0.009668	3.7	0.002769	-199.7	122.1
2 (1.088)	404.80	0.6923	279	0.002470	-163.8	119.2
	399.51	0.4326	174	0.002503	-167.7	119.5
	394.52	0.2751	110	0.002535	-171.5	119.9
	389.54	0.17339	69	0.002567	-175.4	120.2
	384.56	0.10793	42	0.002600	-179.4	120.6
	379.56	0.06557	26	0.002635	-183.6	120.9
	374.37	0.03894	15	0.002671	-188.0	121.2
	369.11	0.02260	8.7	0.002709	-192.6	121.6
[C_2mim][CF_3SO_3]						
$\ln(p^*_{sat}/p^o) = -\frac{74041}{RT_0} - \frac{126379}{R}\left(\frac{1}{T} - \frac{1}{T_0}\right) - \frac{68}{R}\left(\frac{T_0}{T} - 1 - \ln\left(\frac{T}{T_0}\right)\right)$, T_0 = 412.8 K						
1	429.36	0.4524	175	0.002329	-167.6	125.3
	424.38	0.3014	116	0.002356	-171.1	125.6
	419.40	0.2002	76	0.002384	-174.5	125.9
	414.42	0.1317	50	0.002413	-178.1	126.3
	409.43	0.08422	32	0.002442	-181.8	126.6
	404.43	0.05364	20	0.002473	-185.6	126.9
	399.44	0.03339	12	0.002504	-189.6	127.3
	394.45	0.02050	7.6	0.002535	-193.7	127.6
2	431.88	0.5462	211	0.002315	-166.0	125.1
	426.92	0.3745	144	0.002342	-169.2	125.4

	421.93	0.2536	97	0.002370	-172.5	125.8
	416.94	0.1641	62	0.002398	-176.2	126.1
	411.95	0.1054	40	0.002427	-180.0	126.4
	406.96	0.06650	25	0.002457	-183.8	126.8
	401.56	0.04089	15	0.002490	-187.9	127.1
	396.36	0.02462	9.1	0.002523	-192.1	127.5
[C$_2$mim][CH$_3$SO$_3$]						
$\ln(p^*_{\mathrm{sat}}/p^\circ) = -\frac{75179}{RT_0} - \frac{133033}{R}\left(\frac{1}{T} - \frac{1}{T_0}\right) - \frac{68}{R}\left(\frac{T_0}{T} - 1 - \ln\left(\frac{T}{T_0}\right)\right)$, T_0 = 422.3 K						
1	432.41	0.2842	124	0.002313	-170.5	132.3
	427.21	0.1807	78	0.002341	-174.4	132.7
	422.17	0.1154	50	0.002369	-178.1	133.0
	417.17	0.07312	31	0.002397	-182.0	133.4
	412.15	0.04621	20	0.002426	-185.8	133.7
	407.13	0.02872	12	0.002456	-189.8	134.1
	442.27	0.6418	282	0.002261	-163.7	131.7
	432.41	0.2842	124	0.002313	-170.5	132.3
	427.21	0.1807	78	0.002341	-174.4	132.7
	422.17	0.1154	50	0.002369	-178.1	133.0
2	437.28	0.4199	184	0.002287	-167.2	132.0
	432.26	0.2754	120	0.002313	-170.8	132.4
	427.25	0.1784	77	0.002341	-174.5	132.7
	422.21	0.1163	50	0.002368	-178.1	133.0
	417.19	0.07407	32	0.002397	-181.9	133.4
	412.17	0.04633	20	0.002426	-185.8	133.7
	407.17	0.02896	12	0.002456	-189.8	134.1
	402.16	0.01784	7.5	0.002487	-193.9	134.4
3	444.78	0.7698	340	0.002248	-162.1	131.5
	439.86	0.5070	222	0.002273	-165.7	131.8
	434.80	0.3364	147	0.002300	-169.1	132.2
	429.79	0.2229	97	0.002327	-172.6	132.5
	424.75	0.1461	63	0.002354	-176.1	132.9
	419.75	0.09434	40	0.002382	-179.8	133.2
	414.70	0.05870	25	0.002411	-183.8	133.5
	409.69	0.03762	16	0.002441	-187.6	133.9
	406.28	0.02679	11	0.002461	-190.4	134.1
	444.78	0.7698	340	0.002248	-162.1	131.5

[C$_2$mim][BF$_4$]						
$\ln(p^*_{sat}/p^\circ) = -\frac{76640}{RT_0} - \frac{122267}{R}\left(\frac{1}{T} - \frac{1}{T_0}\right) - \frac{62}{R}\left(\frac{T_0}{T} - 1 - \ln\left(\frac{T}{T_0}\right)\right)$, T_0 = 431.6 K						
1	454.31	0.6126	284	0.002201	-163.6	120.9
	449.35	0.4321	201	0.002225	-166.5	121.2
	444.32	0.2991	140	0.002251	-169.5	121.5
	439.34	0.2066	97	0.002276	-172.5	121.8
	434.35	0.1390	67	0.002302	-175.7	122.1
	429.35	0.0891	44	0.002329	-179.1	122.4
	424.38	0.0557	29	0.002356	-182.5	122.7
	419.38	0.0336	20	0.002384	-185.9	123.0
	414.38	0.0191	13	0.002413	-189.2	123.3
2	451.88	0.5682	245	0.002213	-164.9	121.0
	441.86	0.2748	117	0.002263	-171.0	121.6
	431.89	0.1288	54	0.002315	-177.4	122.2
	426.89	0.0887	37	0.002343	-180.5	122.6
	421.89	0.0582	24	0.002370	-184.1	122.9
	416.89	0.0385	16	0.002399	-187.6	123.2
	411.89	0.0242	10	0.002428	-191.5	123.5
[C$_2$mim][PF$_6$]						
$\ln(p^*_{sat}/p^\circ) = -\frac{78196}{R} - \frac{129904}{R}\left(\frac{1}{T} - \frac{1}{T_0}\right) - \frac{74}{R}\left(\frac{T_0}{T} - 1 - \ln\left(\frac{T}{T_0}\right)\right)$, T_0 = 435.2 K						
1	456.79	0.5574	224	0.002189	-165.6	128.3
	451.80	0.3823	153	0.002213	-168.8	128.7
	446.81	0.2640	105	0.002238	-171.9	129.0
	441.84	0.1798	71	0.002263	-175.2	129.4
	436.82	0.1200	47	0.002289	-178.6	129.8
	431.85	0.08051	31	0.002316	-181.9	130.2
	426.85	0.05213	20	0.002343	-185.6	130.5
	421.85	0.03391	13	0.002370	-189.2	130.9
	416.85	0.02170	8.3	0.002399	-193.0	131.3
2	454.34	0.4635	185	0.002201	-167.2	128.5
	450.31	0.3417	136	0.002221	-169.7	128.8
	444.31	0.2159	85	0.002251	-173.6	129.2
	439.29	0.1447	57	0.002276	-177.0	129.6
	434.32	0.09632	38	0.002302	-180.4	130.0
	429.33	0.06477	25	0.002329	-183.8	130.3
	424.34	0.04285	17	0.002357	-187.3	130.7

	419.34	0.02766	11	0.002385	-190.9	131.1
	414.05	0.01710	6.5	0.002415	-195.0	131.5

[C_2mim][B(CN)$_4$]

$$\ln(p^*_{\mathrm{sat}}/p^\circ) = -\frac{74506}{R} - \frac{124865}{R}\left(\frac{1}{T} - \frac{1}{T_0}\right) - \frac{37}{R}\left(\frac{T_0}{T} - 1 - \ln\left(\frac{T}{T_0}\right)\right),\ T_0 = 403.9\ \mathrm{K}$$

1	427.74	0.4085	169	0.002338	-167.9	124.0
	422.75	0.2910	120	0.002365	-170.8	124.2
	417.73	0.2006	82	0.002394	-174.0	124.4
	412.75	0.1326	54	0.002423	-177.5	124.5
	407.76	0.08151	33	0.002452	-181.5	124.7
	402.76	0.04971	20	0.002483	-185.7	124.9
	397.77	0.03122	12	0.002514	-189.6	125.1
	392.77	0.01941	7.7	0.002546	-193.6	125.3
	387.78	0.01208	4.8	0.002579	-197.6	125.5
	382.78	0.007363	2.9	0.002612	-201.8	125.6
2	425.24	0.3257	134	0.002352	-169.9	124.1
	420.23	0.2227	91	0.002380	-173.1	124.3
	415.23	0.1534	62	0.002408	-176.2	124.4
	410.27	0.1020	41	0.002437	-179.7	124.6
	405.30	0.06620	27	0.002467	-183.3	124.8
	400.32	0.04145	17	0.002498	-187.2	125.0
	395.33	0.02583	10	0.002530	-191.2	125.2
	390.33	0.01572	6.2	0.002562	-195.4	125.4
	385.33	0.009758	3.8	0.002595	-199.4	125.6
	380.33	0.005939	2.3	0.002629	-203.6	125.7
3	427.77	0.4372	181	0.002338	-167.4	124.0
	422.76	0.3076	126	0.002365	-170.4	124.2
	417.74	0.2114	86	0.002394	-173.5	124.4
	412.71	0.1379	56	0.002423	-177.1	124.5
	407.69	0.08929	36	0.002453	-180.8	124.7
	402.67	0.05332	21	0.002483	-185.1	124.9
	397.65	0.03454	14	0.002515	-188.8	125.1
	392.63	0.02083	8.2	0.002547	-193.1	125.3
	387.61	0.01169	4.6	0.002580	-197.9	125.5
	382.60	0.007601	3.0	0.002614	-201.5	125.7

[C_2mim][SCN]

$$\ln(p^*_{\mathrm{sat}}/p^\circ) = -\frac{75773}{R} - \frac{142145}{R}\left(\frac{1}{T} - \frac{1}{T_0}\right) - \frac{62}{R}\left(\frac{T_0}{T} - 1 - \ln\left(\frac{T}{T_0}\right)\right),\ T_0 = 413.2\ \mathrm{K}$$

1	434.90	0.4186	202	0.002299	-166.5	140.8
	429.90	0.2750	132	0.002326	-170.0	141.1
	424.89	0.1747	83	0.002354	-173.8	141.4
	419.88	0.1083	51	0.002382	-177.9	141.7
	414.86	0.06644	31	0.002410	-182.0	142.0
	409.86	0.03989	19	0.002440	-186.3	142.4
	404.85	0.02443	11	0.002470	-190.4	142.7
	399.85	0.01436	6.6	0.002501	-194.9	143.0
	394.85	0.008275	3.8	0.002533	-199.5	143.3
2	432.33	0.3577	172	0.002313	-167.8	141.0
	427.35	0.2155	103	0.002340	-172.1	141.3
	422.33	0.1318	63	0.002368	-176.2	141.6
	417.33	0.08196	39	0.002396	-180.2	141.9
	412.33	0.05079	24	0.002425	-184.2	142.2
	407.33	0.03176	15	0.002455	-188.2	142.5
	402.32	0.01868	8.7	0.002486	-192.7	142.8
	397.31	0.01071	4.9	0.002517	-197.3	143.1
	392.30	0.006427	2.9	0.002549	-201.6	143.4

[C_2mim][C_2SO_4]

$$\ln(p^*_{\mathrm{sat}}/p^\circ) = -\frac{80852}{R} - \frac{143623}{R}\left(\frac{1}{T} - \frac{1}{T_0}\right) - \frac{73}{R}\left(\frac{T_0}{T} - 1 - \ln\left(\frac{T}{T_0}\right)\right),\ T_0 = 421.2\ \mathrm{K}$$

1	432.38	0.06739	27	0.002313	-183.1	148.2
	429.94	0.05356	22	0.002326	-185.0	148.4
	427.41	0.04237	17	0.002340	-187.0	148.5
	424.90	0.03309	13	0.002353	-189.1	148.7
	422.25	0.02560	10	0.002368	-191.2	148.9
	419.90	0.02091	8.4	0.002382	-192.9	149.1
	417.36	0.01598	6.4	0.002396	-195.2	149.3
	415.04	0.01268	5.1	0.002409	-197.1	149.4
	412.81	0.01036	4.1	0.002422	-198.8	149.6
	410.02	0.007869	3.1	0.002439	-201.1	149.8
	407.40	0.005943	2.3	0.002455	-203.5	150.0
2	437.74	0.1086	44	0.002284	-179.1	147.8
	434.95	0.08459	34	0.002299	-181.2	148.0
	431.39	0.06076	25	0.002318	-183.9	148.2
	426.45	0.03863	16	0.002345	-187.8	148.6
	421.45	0.02399	10	0.002373	-191.8	149.0

	416.47	0.01470	5.9	0.002401	-195.9	149.3
	411.53	0.009119	3.6	0.002430	-199.9	149.7
	406.45	0.005384	2.1	0.002460	-204.3	150.1
[C2mim][C1SO4]						
$\ln(p^*_{\mathrm{sat}}/p^\circ) = -\frac{80351}{R} - \frac{135155}{R}\left(\frac{1}{T} - \frac{1}{T_0}\right) - \frac{65}{R}\left(\frac{T_0}{T} - 1 - \ln\left(\frac{T}{T_0}\right)\right)$, T_0 = 442.0 K						
1	457.25	0.25611	110	0.002187	-171.5	134.2
	452.23	0.17013	73	0.002211	-174.9	134.5
	447.23	0.11302	48	0.002236	-178.4	134.8
	442.21	0.07569	32	0.002261	-181.8	135.1
	437.21	0.05036	21	0.002287	-185.2	135.5
	432.19	0.03307	14	0.002314	-188.7	135.8
	427.17	0.02143	8.9	0.002341	-192.4	136.1
	422.15	0.01371	5.7	0.002369	-196.2	136.4
2	464.79	0.44427	193	0.002152	-166.8	133.7
	459.76	0.30388	131	0.002175	-170.0	134.0
	454.75	0.20727	89	0.002199	-173.3	134.3
	449.79	0.14146	60	0.002223	-176.5	134.7
	444.78	0.09447	40	0.002248	-179.9	135.0
	439.76	0.06265	26	0.002274	-183.4	135.3
	434.74	0.04127	17	0.002300	-186.9	135.6
	429.71	0.02651	11	0.002327	-190.6	136.0
	424.73	0.01706	7.1	0.002354	-194.3	136.3
[C2mim][C4SO4]						
$\ln(p^*_{\mathrm{sat}}/p^\circ) = -\frac{79781}{R} - \frac{144369}{R}\left(\frac{1}{T} - \frac{1}{T_0}\right) - \frac{83}{R}\left(\frac{T_0}{T} - 1 - \ln\left(\frac{T}{T_0}\right)\right)$, T_0 = 437.9 K						
1	459.79	0.508955	202	0.002175	-166.5	142.6
	454.80	0.335185	132	0.002199	-170.0	143.0
	449.78	0.219541	86	0.002223	-173.6	143.4
	444.76	0.143206	56	0.002248	-177.2	143.8
	439.75	0.092342	36	0.002274	-180.8	144.2
	434.72	0.058632	23	0.002300	-184.7	144.6
	429.72	0.037376	14	0.002327	-188.5	145.0
	424.64	0.022847	8.7	0.002355	-192.6	145.5
	419.63	0.014198	5.4	0.002383	-196.6	145.9
2	462.31	0.614672	244	0.002163	-164.9	142.3
	457.32	0.409215	162	0.002187	-168.3	142.8
	452.32	0.271785	107	0.002211	-171.8	143.2

	447.29	0.177676	69	0.002236	-175.3	143.6
	442.31	0.116201	45	0.002261	-178.9	144.0
	437.23	0.074952	29	0.002287	-182.6	144.4
	432.20	0.047748	18	0.002314	-186.4	144.8
	427.21	0.030756	12	0.002341	-190.1	145.3
	422.17	0.018083	6.9	0.002369	-194.6	145.7
	417.16	0.010804	4.1	0.002397	-198.9	146.1
	412.16	0.006773	2.5	0.002426	-202.8	146.5
[C_2mim][C_8SO_4]						
$\ln(p^*_{\text{sat}}/p^\circ) = -\frac{82288}{R} - \frac{156923}{R}\left(\frac{1}{T} - \frac{1}{T_0}\right) - \frac{99}{R}\left(\frac{T_0}{T} - 1 - \ln\left(\frac{T}{T_0}\right)\right)$, T_0 = 447.8 K						
1	467.50	0.406055	147	0.002139	-169.1	155.0
	462.28	0.260072	94	0.002163	-172.8	155.5
	457.26	0.1666	60	0.002187	-176.6	156.0
	452.27	0.1066	38	0.002211	-180.3	156.5
	447.24	0.0674	24	0.002236	-184.2	157.0
	442.22	0.0422	15	0.002261	-188.1	157.5
	437.18	0.02610	9.2	0.002287	-192.2	158.0
	432.17	0.01550	5.4	0.002314	-196.6	158.5
	427.16	0.00930	3.2	0.002341	-200.9	159.0
	420.54	0.00476	1.6	0.002378	-206.5	159.6
2	472.43	0.61960	226	0.002117	-165.5	154.5
	467.33	0.401349	146	0.002140	-169.2	155.0
	462.33	0.262528	95	0.002163	-172.8	155.5
	457.31	0.168562	60	0.002187	-176.5	156.0
	452.29	0.107018	38	0.002211	-180.3	156.5
	447.24	0.067166	24	0.002236	-184.2	157.0
	442.25	0.041352	15	0.002261	-188.3	157.5
	436.93	0.024863	8.7	0.002289	-192.6	158.0
	433.02	0.017013	5.9	0.002309	-195.8	158.4
[C_2mim][$(C_2F_5)_3PF_3$]						
$\ln(p^*_{\text{sat}}/p^\circ) = -\frac{70697}{R} - \frac{118278}{R}\left(\frac{1}{T} - \frac{1}{T_0}\right) - \frac{109}{R}\left(\frac{T_0}{T} - 1 - \ln\left(\frac{T}{T_0}\right)\right)$, T_0 = 373.4 K						
1	397.59	0.4835	123	0.00252	-170.6	115.6
	392.60	0.3171	80	0.00255	-174.2	116.2
	387.61	0.2077	52	0.00258	-177.7	116.7
	382.62	0.1320	33	0.00261	-181.5	117.3
	377.66	0.08199	20	0.00265	-185.6	117.8

	372.69	0.04959	12	0.00268	-189.8	118.4
	367.71	0.02975	7.3	0.00272	-194.1	118.9
	362.71	0.01715	4.2	0.00276	-198.7	119.4
	357.72	0.00996	2.4	0.00280	-203.3	120.0
	352.73	0.005533	1.3	0.00284	-208.3	120.5
2	395.08	0.3996	101	0.00253	-172.2	115.9
	390.11	0.2586	65	0.00256	-175.9	116.5
	385.14	0.1638	41	0.00260	-179.7	117.0
	380.20	0.1036	26	0.00263	-183.6	117.5
	375.23	0.06385	16	0.00267	-187.7	118.1
	370.25	0.03843	9.4	0.00270	-191.9	118.6
	365.26	0.02281	5.6	0.00274	-196.3	119.2
	360.27	0.01325	3.2	0.00278	-200.9	119.7
	355.28	0.007454	1.8	0.00281	-205.8	120.3
	350.29	0.004186	1.0	0.00285	-210.6	120.8
[C_2mim][$(C_2H_5O)_2PO_2$]						
$\ln(p_{\mathrm{sat}}^{*}/p^{\circ}) = -\frac{70893}{R} - \frac{136589}{R}\left(\frac{1}{T} - \frac{1}{T_0}\right) - \frac{81}{R}\left(\frac{T_0}{T} - 1 - \ln\left(\frac{T}{T_0}\right)\right)$, T_0 = 392.9 K						
1	409.51	0.5334	199	0.002442	-166.6	135.2
	404.47	0.3406	127	0.002472	-170.3	135.7
	399.50	0.2064	76	0.002503	-174.6	136.1
	394.49	0.1210	44	0.002535	-179.1	136.5
	389.51	0.0697	25	0.002567	-183.7	136.9
	384.56	0.0411	15	0.002600	-188.1	137.3
	379.55	0.02328	8.4	0.002635	-192.9	137.7
	374.57	0.01382	4.9	0.002670	-197.3	138.1
2	412.02	0.6925	260	0.002427	-164.4	135.0
	407.00	0.4249	158	0.002457	-168.5	135.5
	402.01	0.2590	96	0.002488	-172.6	135.9
	397.00	0.1573	58	0.002519	-176.8	136.3
	392.01	0.09372	34	0.002551	-181.2	136.7
	387.04	0.05466	20	0.002584	-185.7	137.1
	382.04	0.03152	11	0.002618	-190.4	137.5
	377.05	0.01800	6.5	0.002652	-195.1	137.9
[C_2mim][4-CH_3-Ph-SO_3]						
$\ln(p_{\mathrm{sat}}^{*}/p^{\circ}) = -\frac{82263}{R} - \frac{149823}{R}\left(\frac{1}{T} - \frac{1}{T_0}\right) - \frac{91}{R}\left(\frac{T_0}{T} - 1 - \ln\left(\frac{T}{T_0}\right)\right)$, T_0 = 360.5 K						
1	479.65	0.5649	221	0.002085	-165.7	148.1

	474.64	0.3849	150	0.002107	-168.9	148.5
	469.63	0.2578	100	0.002129	-172.3	149.0
	464.57	0.1710	66	0.002153	-175.8	149.4
	459.57	0.1119	43	0.002176	-179.3	149.9
	454.55	0.07227	28	0.002200	-183.0	150.4
	449.53	0.04730	18	0.002225	-186.6	150.8
	444.52	0.03010	11	0.002250	-190.4	151.3
	439.50	0.01925	7.2	0.002275	-194.2	151.7
2	482.18	0.6860	269	0.002074	-164.1	147.8
	477.13	0.4696	183	0.002096	-167.3	148.3
	472.13	0.3135	122	0.002118	-170.7	148.8
	467.08	0.2096	81	0.002141	-174.1	149.2
	462.07	0.1385	53	0.002164	-177.5	149.7
	457.04	0.09002	34	0.002188	-181.2	150.1
	452.04	0.05896	22	0.002212	-184.7	150.6
	447.05	0.03803	14	0.002237	-188.4	151.0
	442.02	0.02416	9.1	0.002262	-192.2	151.5
$[C_2mim][C(CN)_3]$						
$\ln(p^*_{\rm sat}/p^\circ) = -\frac{77427}{R} - \frac{126027}{R}\left(\frac{1}{T} - \frac{1}{T_0}\right) - \frac{56}{R}\left(\frac{T_0}{T} - 1 - \ln\left(\frac{T}{T_0}\right)\right)$, T_0 = 423.2 K						
1	447.49	0.4273	191	0.002235	-166.9	124.7
	442.48	0.2941	131	0.002260	-170.1	124.9
	437.47	0.2035	90	0.002286	-173.2	125.2
	432.45	0.1377	61	0.002312	-176.5	125.5
	427.44	0.09106	40	0.002340	-179.9	125.8
	422.42	0.05955	26	0.002367	-183.5	126.1
	417.41	0.03849	17	0.002396	-187.2	126.4
	412.40	0.02487	11	0.002425	-190.9	126.6
	407.41	0.01573	6.7	0.002455	-194.8	126.9
	402.40	0.01039	4.4	0.002485	-198.2	127.2
2	444.93	0.3568	159	0.002248	-168.4	124.8
	439.94	0.2416	107	0.002273	-171.7	125.1
	434.94	0.1635	72	0.002299	-175.0	125.4
	429.95	0.1103	48	0.002326	-178.3	125.6
	424.96	0.07358	32	0.002353	-181.7	125.9
	419.97	0.04833	21	0.002381	-185.3	126.2
	414.99	0.03156	14	0.002410	-188.9	126.5

	410.00	0.02037	8.7	0.002439	-192.6	126.8
	405.01	0.01314	5.6	0.002469	-196.3	127.0
	400.03	0.008172	3.5	0.002500	-200.3	127.3
[C$_2$mim][NTf$_2$]						
$\ln(p_{sat}^{*}/p^{\circ}) = -\frac{70018}{R} - \frac{118558}{R}\left(\frac{1}{T} - \frac{1}{T_0}\right) - \frac{56}{R}\left(\frac{T_0}{T} - 1 - \ln\left(\frac{T}{T_0}\right)\right)$, T_0 = 378.2 K						
1	392.24	0.2723	82	0.002549	-174.0	117.8
	387.24	0.1718	51	0.002582	-177.8	118.1
	382.23	0.1069	32	0.002616	-181.8	118.3
	377.24	0.06569	19	0.002651	-185.9	118.6
	372.24	0.03977	12	0.002686	-190.2	118.9
	367.24	0.02374	6.9	0.002723	-194.5	119.2
	362.25	0.01399	4.0	0.002761	-199.0	119.5
2	394.67	0.3400	103	0.002534	-172.1	117.6
	389.72	0.2163	65	0.002566	-175.9	117.9
	384.78	0.1365	41	0.002599	-179.8	118.2
	379.76	0.08408	25	0.002633	-183.9	118.5
	374.77	0.05148	15	0.002668	-188.0	118.8
	369.76	0.03082	9.0	0.002704	-192.3	119.0
	364.75	0.01823	5.3	0.002742	-196.7	119.3

[a] The combined expanded uncertainties are U_c(T) = 0.02 K, $U_r(df{\cdot}dt^{-1})$ = 0.01 for confidence level = 0.95, $k \approx 2$.
[b] From reference [1]
[c] Calculated in this work from the primary data on the frequency shift velocities df/dt

Table S2: PC-SAFT results for the correlation of LLE, VLE or IDAC data of binary mixture containing the [C_2mim]-IL systems under investigation, and the respective binary interaction parameters. ARD% and AAD for the correlations are given for the two parameter sets of the [C_2mim]-ILs in Table 4 (use of vapor pressure and liquid density) and Table 5 (liquid density only).

Organic Compound	System	*Table 4* k_{ij}	*Table 4* *AAD*	*Table 4* *ARD%*	*Table 5* k_{ij}	*Table 5* *AAD*	*Table 5* *ARD%*
				[C_2mim][NTf_2]			
Water	LLE	-0.045	0.056	8.122	0.005	0.050	7.258
CO_2	VLE	0.200	38.99	28.64	0.100	25.59	35.85
				[C_2mim][SCN]			
Water	IDAC	-0.090	0.01	3.29	-0.145	0.01	4.84
CO_2	VLE	0.120	0.10	6.72	0.270	0.09	7.80
				[C_2mim][CF_3CO_2]			
Water	IDAC	-0.135	0.01	6.81	-0.205	0.003	2.02
CO_2	VLE	0.125	0.38	73.57	0.200	0.46	67.75
				[C_2mim][CF_3SO_3]			
Water	VLE	0.037	0.02	26.92	-0.040	0.02	23.16
Methanol	IDAC	-0.103	0.03	3.75	-0.160	0.06	8.45
				[C_2mim][$(C_2H_5O)_2PO_2$]			
CO_2	VLE	0.360	17.03	59.58	0.220	1.90	4.09
Hexane	IDAC	0.008	5.56	7.88	0.190	4.24	6.17
Pentane	IDAC	0.030	2.38	5.80	0.193	2.16	5.19
				[C_2mim][PF_6]			
CO_2	VLE	0.088	139.21	39.24	0.088	181.00	34.58
H_2S	VLE	0.002	1.02	10.76	0.018	0.46	5.49
				[C_2mim][BF_4]			
Water	VLE	0.030	0.10	36.68	-0.067	0.05	20.21
Water	IDAC	0.030	0.003	0.59	-0.067	0.002	0.38
Benzene	VLE	-0.005	0.001	2.88	0.015	0.002	5.92
				[C_2mim][$B(CN)_4$]			
Water	IDAC	0.024	0.02	1.08	-0.030	0.08	5.24
CO_2	VLE	0.140	3.19	14.49	0.190	2.91	11.98
				[C_2mim][$C(CN)_3$]			
Water	IDAC	-0.017	-0.02	0.07	-0.050	0.04	4.76
CO_2	VLE	0.075	0.08	0.21	0.235	0.17	7.39
				[C_2mim][CH_3SO_3]			
Water	VLE	-0.070	0.002	35.19	-0.200	0.002	21.41
Water	IDAC	-0.225	0.003	3.15	-0.340	0.002	3.21
CO_2	VLE	0.150	4.49	16.42	0.250	4.16	14.23
				[C_2mim][$(C_2F_5)_3PF_3$]			

Water	IDAC	0.150	0.150	0.452	0.150	8.754	0.150
CO_2	VLE	0.160	0.160	0.859	0.160	12.458	0.190
			$[C_2mim][4\text{-}CH_3\text{-}Ph\text{-}SO_3]$				
Methanol	IDAC	-0.060	0.005	1.86	-0.110	0.004	1.35
Ethanol	IDAC	0.063	0.001	0.15	0.042	0.001	0.13
1-Propanol	IDAC	0.080	0.01	2.15	0.088	0.02	2.56
2-Propanol	IDAC	0.105	0.02	1.77	0.097	0.02	2.42
1-Butanol	IDAC	0.063	0.01	0.84	0.043	0.01	1.29

References

(1) Zaitsau, D. H.; Fumino, K.; Emel'yanenko, V. N.; Yermalayeu, A. V.; Ludwig, R.; Verevkin, S. P. Structure-property relationships in ionic liquids: a study of the anion dependence in vaporization enthalpies of imidazolium-based ionic liquids. *Chemphyschem: a European journal of chemical physics and physical chemistry* **2012**, *13*, 1868–1876.
